# CX3CR1 But Not CCR2 Expression Is Required for the Development of Autoimmune Peripheral Neuropathy in Mice

**DOI:** 10.3389/fimmu.2021.720733

**Published:** 2021-08-16

**Authors:** Oladayo Oladiran, Xiang Qun Shi, Sylvie Fournier, Ji Zhang

**Affiliations:** ^1^ The Alan Edwards Centre for Research on Pain, McGill University, Montreal, QC, Canada; ^2^ Department of Microbiology & Immunology, McGill University, Montreal, QC, Canada; ^3^ Department of Neurology & Neurosurgery, McGill University, Montreal, QC, Canada; ^4^ Faculty of Dentistry, McGill University, Montreal, QC, Canada

**Keywords:** macrophages, CD8^+^ T cells, autoimmune peripheral neuropathy, CX3CR1, CCR2, apoptosis, phagocytosis

## Abstract

One hallmark of Guillain-Barre syndrome (GBS), a prototypic autoimmune peripheral neuropathy (APN) is infiltration of leukocytes (macrophages and T cells) into peripheral nerves, where chemokines and their receptors play major roles. In this study, we aimed to understand the potential contribution of chemokine receptors CCR2 and CX3CR1 in APN by using a well-established mouse model, B7.2 transgenic (L31) mice, which possesses a predisposed inflammatory background. We crossbred respectively CCR2KO and CX3CR1KO mice with L31 mice. The disease was initiated by partial ligation on one of the sciatic nerves. APN pathology and neurological function were evaluated on the other non-ligated sciatic nerve/limb. Our results revealed that L31/CX3CR1KO but not L31/CCR2KO mice were resistant to APN. CX3CR1 is needed for maintaining circulating monocyte and CD8^+^ T cell survival. While migration of a significant number of activated CD8^+^ T cells to peripheral nerves is essential in autoimmune response in nerve, recruitment of monocytes into PNS seems optional. Disease onset is independent of CCR2 mediated blood-derived macrophage recruitment, which can be replaced by compensatory proliferation of resident macrophages in peripheral nerve. CX3CR1 could also contribute to APN *via* its critical involvement in maintaining nerve macrophage phagocytic ability. We conclude that blockade of CX3CR1 signaling may represent an interesting anti-inflammatory strategy to improve therapeutic management for GBS patients.

## Introduction

Guillain Barre syndrome (GBS) is an acute inflammatory, demyelinating disease of the peripheral nervous system (PNS). It is the most common cause of acute paralysis with an incidence of 1 to 2 cases per 100,000 population per year (1, 2). Current treatment options include intravenous immunoglobulin and plasmapheresis which limit consequential peripheral nerve demyelination and axonal injury. However, mortality occurs in 5-10% patients and 20% are left with residual deficits that affects everyday living (3). Pathologically, GBS is characterized by demyelination and/or axonal loss and leukocyte infiltration. While pathological changes have been well documented, molecular, and cellular mechanisms remains poorly understood. Limited collective evidence from human and animal studies points to a macrophage-induced demyelination associated with intense T-cell and plasma cell infiltration into the PNS (2, 4). Leukocyte adhesion and extravasation with multiple well-defined steps are mediated by some adhesion molecules and chemokines (5, 6). Signaling between chemokines and their cognate receptors is of particular interest since they can selectively traffic leukocyte subsets.

Chemokines are low molecular weight cytokines involved in chemotaxis and activation of leukocytes (7). Despite many publications on chemokines and receptors in human diseases in the past 2 decades, the literature on chemokine biology of GBS remains relatively sparse. The putative roles of specific chemokines and their receptors in GBS pathogenesis are supported by several observations from both human and animal studies. CCL2-CCR2, CXCL10-CXCR3, and CCL5-CCR5 are the most implicated chemokine pairs. They were upregulated in nerves of rat experimental allergic neuritis (EAN) (8–10). Similar expression pattern was found in cerebrospinal fluid (CSF) (11), blood (9), and sural nerve biopsy of GBS patients (12). Compared with observational studies, functional studies of chemokine mediated signaling in GBS are rare. Additional investigations are needed to further confirm causal relationship between chemokines/receptors and demyelinating inflammatory peripheral neuropathy.

Blood monocytes exist as two phenotypes- inflammatory and resident/patrolling, each of which expresses distinct markers. Patrolling monocytes are CCR2^low^ CX3CR1^high^ and they predominantly have homeostatic function (13). Inflammatory monocytes on the other hand, display CCR2^high^ and CX3CR1^low^ expression (13). They are rapidly recruited to the sites of nerve injury and inflammation in a CCL2/CCR2 dependent manner where they corroborate resident nerve macrophages to facilitate wound repair and healing (14, 15). CCR2 is important for the egress of monocytes from the bone marrow to the blood as well as their migration into inflamed tissue. Mice devoid of the CCR2 gene expression exhibit markedly reduced recruitment of monocytes to sites of inflammation (14). CX3CR1, is also an important chemokine receptor for monocytes and macrophages. It mediates monocyte survival and recruitment under certain conditions. It also modulates macrophage/microglial functions in health and disease (16–18).

As a continuation of our previous study (19) describing the importance of nerve macrophage activation in APN by using B7.2 (L31) transgenic mice, here, we aim to dissect distinct contribution of chemokine receptors CX3CR1 and CCR2 on monocytes/macrophages and CD8^+^ T cells in the genesis of APN. Our results clearly demonstrated that CX3CR1 but not CCR2 signaling has critical roles in developing APN, as CX3CR1, but not CCR2 deficiency protects mice from systemic and nerve inflammation, demyelination, and axonal loss. As a consequence, L31/CX3CR1KO mice preserve their normal neurological functions. We also revealed the critical involvement of CX3CR1 in the survival of circulating monocytes and CD8^+^ T cells. However, CCR2-mediated blood derived macrophage recruitment to peripheral nerve could be replaced by resident macrophage cell proliferation. In the presence of activated CD8^+^ T cells, CX3CR1^+^ resident macrophages, having enhanced phagocytic ability, can initiate APN in L31 mice.

## Materials and Methods

### Animals

B7.2 transgenic (L31) mice were generated and interbred as previously described (20, 21). In these mice, the B7.2 cDNA is under the transcriptional control of MHC class I promoter and Igµ enhancer. Mice spontaneously develop APN between 2-6 months. CX3CR1KO (005582) and CCR2KO (004999) mice were purchased from The Jackson Laboratory. CX3CRKO mice were crossed with L31 mice for more than 6 generations to obtain L31 (B7.2^+/-^)/CX3CR1^-/-^ mice. L31 (B7.2^+/-^)/CCR2^-/-^ mice were generated by crossing CCR2KO mice to L31 mice (> 6 generations). Knockout status was confirmed by genotyping before experimentation. C57BL/6 mice bred and housed in the same facility were used as controls (wild type mice). All data were collected and pooled from both male and female mice, since no significant difference in disease incidence, severity and pathology, nor major/significant difference in circulating immune cells were observed between sexes. All procedures were in accordance with the guidelines of the Canadian Council on Animal Care and approved by the animal care committee of McGill University.

### Nerve Injury Model

Asides the spontaneous development of APN by L31 mice, we previously reported (22) that an injury to one of peripheral nerves accelerated the development of APN in other non-injured nerves in L31 mice. In brief, partial sciatic nerve ligation (PSNL) was performed in L31 mice. Left sciatic nerve was exposed at high thigh level after mice were anesthetized by 3% isoflurane, an 8-0 silk suture was inserted into the nerve with a 3/8 curved, reversed-cutting mini-needle and tightly ligated, one third to one half of the dorsal part of sciatic was trapped in ligature. The wound was closed by a 4-0 skin suture. All data presented in this study are from the L31-PSNL model. All experimental assessments were performed on the uninjured right sciatic nerve and right limb.

### Behavior Test

We assessed mouse neurological functions with different motor and sensory behavioral tests to determine their disability and recovery (n= 10-15/group). The behavioral experimenter was blinded to the genetic and treatment status of the animals.

*Clinical scores* as previously described (21) was used to evaluate motor deficits; 0 – normal; 1 – reduced tonus of tail and/or limp tail; 2 – paresis of contralateral hind limb, staying in clasping or outstretching position when lifted by the tail; 3 – paresis of both hind limbs, staying in clasping or outstretching position when lifted by the tail; 4 – paralysis or splaying of contralateral hind limb; 5 – paralysis or splaying of both hind limbs; 6 – moribund or death. Data generated in this study was based on the evaluation of contralateral limb only. Positive signs appearing on the ipsilateral limb were excluded to avoid interference of PSNL. Clinical scores 3 and 5 were excluded because they record signs on both ipsilateral and contralateral limbs. Only positive signs on the contralateral limb were taken into consideration, thus score 2 and 4 were included in our evaluation.

*Grip strength test* was performed to test neuromuscular function. Test was carried out by assessing grasping applied by a mouse using a grip strength meter (Stoelting Co). Mice were held at the base of the tail and lowered over the grid while ensuring that the torso was parallel with the grid with both the forepaws and hind paws attached to the grid. Mice were then gently pulled back by the tail and the maximal grip strength was recorded in grams. Procedure was repeated twice, and values were averaged. Mice were returned to their home cages for approximately 20 mins between each measurement.

*Von Frey test* as previously described (23) was performed to test paw sensitivity to mechanical stimuli. Mice were placed on a metal mesh floor with small Plexiglas cubicles for at least one hour for habituation before testing. A set of eight calibrated monofilaments (Stoelting) were applied to the plantar surface of the hind paw until they bent, the 50% threshold to withdraw was determined by an average of two tests separated by at least 1 hour. An increase in threshold suggests mechanical hyposensitivity.

*Acetone test* was performed to evaluate sensitivity to cold stimulation. A drop of acetone, approx. 25 µL, was applied to the plantar surface of the hind paw. The duration of acetone evoked behaviors (flinching, licking, or biting) following application of one single drop of acetone within 1 min observation was recorded. An increase in the duration of above-mentioned pain like behavior indicates cold hypersensitivity.

### Histological Analyses

Axon and myelin structure was visualized using toluidine blue staining on semi-thin nerve cross-sections. Mice (n=5-8/group) were perfused with a fixative solution (0.5% PFA + 2.5% glutaraldehyde + 0.1 M phosphate buffer) at the end of the experiment. Sciatic nerves were removed and post-fixed in the same fixative overnight. Nerve samples were processed with osmium tetroxide, dehydrated, and embedded in epon kit. Sciatic nerves were sectioned at 0.5 µm using an ultramicrotome (Leica-Reichert) with a diamond knife (Diatome Switzerland). Sections were then stained with toluidine blue for 40 secs at 60°C. Images were obtained by using an Olympus BX51 microscope equipped with a color digital camera. The severity of nerve damage was assessed by measuring demyelinated area over total nerve cross section surface (n=5-8/group). In the area where nerve structure was generally maintained, 100 myelinated axons per section (5-8 nerves/group) were randomly selected to measure myelin and axon area by using ImageJ software (NIH), which allows the assessment of microinjury in sciatic nerves.

### Flow Cytometry

Single cell suspension from blood and sciatic nerve of (n=5-10/group) was prepared as previously described (24). In brief, 50 µl whole blood was collected from sub-mandibular vein of mice and kept in pre-cold Alsevier’s solution (Gibco) to prevent coagulation. After a brief spin down and removal of the supernatant, erythrocytes were lysed by incubating samples with ACK lysing buffer (Thermo Fisher Scientific) at room temperature for 5 min. Approximately 2 cm-long segments of sciatic was collected following a quick perfusion with 50 µl cold saline. Samples were diced into small pieces and digested by collagenase IV (1.6 mg/ml, Sigma-Aldrich) in 1x HBSS, then passed through a 70 µm cell strainer to obtain the single cell suspension. Following several washes with 1xHBSS, Fc receptors were blocked with 2.4 G2 blocking buffer for 30 mins at 4°C. Samples were then stained with specific fluorochrome-conjugated antibodies for 30 min at 4°C. Data was acquired with FACS Canto II (BD), and analyzed by using flowjo software. Detailed information of antibodies used in the study is listed in **Table 1**.

**Table 1:** List of antibodies used for FACS.

Antibody	Dilution	Source	Catalog number	Clone
CD8	1:50	eBioscience	17-0081-83, 46-0081-82	53-6.7
CD11b	1:50	BD	552850	M1/70
CD45	1:50	Biolegend	103116	30-F11
CD62L	1:50	eBioscience	11-0621-85	MEL-14
CD86	1:50	eBioscience	12-0862-82	GL1
CD115	1:50	eBioscience	53-1152-82	AFS98
CCR2	1:50	Biolegend	150604	SA203G11
CX3CR1	1:50	Biolegend	149005	SA011F11
F4/80	1:50	eBioscience	45-4801-82	BM8
Ki67	1:50	Biolegend	652404	SOIA15
CD44	1:50	Biolgend	103030	1M7
CD43	1:50	Biolgend	121220, 121207	1B11
Annexin V/7AAD kit	1:25	Biolgend	640934	

### Total RNA Extraction and RT-PCR

Mice (n=5-6/group) were sacrificed at the end of experiments to harvest sciatic nerve. Total RNA was extracted by using TRIzol reagent (Ambion Life Technologies). In brief, samples were homogenized in trizol with 0.2 mm glass beads (Sigma-Aldrich) by using Precellys 24 tissue homogenizer (Bertin technologies) at 6500 rpm for 30 secs. Chloroform and isopropanol were then added to remove total protein and genomic DNA, respectively. After washing by 75% ethanol, total RNA was resolved in DEPC treated RNase-free H_2_O. The purity and concentration of RNA was assessed using Nanodrop 2000 (Thermofisher Scientific). 1 µg of total RNA was added into each reverse transcription system, which contains superscript IV reverse transcriptase (Invitrogen), Oligo-dt (18mer) and dNTP (Invitrogen). Real-time quantitative PCR (qPCR) reactions were processed with a Rotor-Gene Q real-time PCR cycler (Qiagen) using SYBR Green mix (Qiagen). The levels of target genes were normalized against the housekeeping gene GAPDH and interpreted using the comparative Ct method. qPCR primers were designed based on gene sequence from GeneBank database on NCBI and synthesized by Integrated DNA Technologies. Primer sequences are listed in **Table 2**.

**Table 2:** List of primers used for RT-PCR.

Primers	Forward	Reverse
GAPDH	GTGAAGGTCGGTGTGAAC	AATCTCCACTTTGCCACTG
IFN-γ	TCCACATCTATGCCACTTGAG	CTGAGACAATGAACGCTACACA
IL-1β	CTATACCTGTCCTGTGTA	GCTCTTGACTTCTATCTTG
IL-6	CTGAAACTTCCAGAGATAC	TTCATGTACTCCAGGTAG
TNF-α	TTCTGTCTACTGAACTTC	CCATAGAACTGATGAG

### Nerve Macrophage Isolation and Phagocytosis Assay

Sciatic nerves were harvested from mice (n=6-10/group), and minced in RPMI + 1% penicillin/streptomycin, and then incubated in RPMI + 1% penicillin/streptomycin + collagenase D for 40 min at 37°C with occasional agitation. Tissue was later passed through a 70 µM filter and centrifuged. Thereafter, cells were washed and resuspended in RPMI + 1% penicillin/streptomycin + 10% FBS and immediately plated in a 96 well plate. To isolate macrophages, cells were cultured for 24 hours, after which culture medium was replaced. Cells were later incubated with fluorescent beads (Invitrogen, F8775) for 2 hours and fluorescent intensity was quantified on a microplate reader (Molecular devices).

### Statistical Analysis

Data are presented as mean ± SEM and analyzed using the Graph Pad Prism software. In general, an unpaired Student’s t-test was used for single comparisons between groups, and a two-way ANOVA, followed by Bonferroni’s *post hoc* analysis tests for multiple comparisons. Differences were considered significant at *p* < 0.05.

## Results

### CX3CR1 and CCR2 Are Highly Expressed in the Blood and Nerves of Diseased L31 Mice

**Figure 1:**
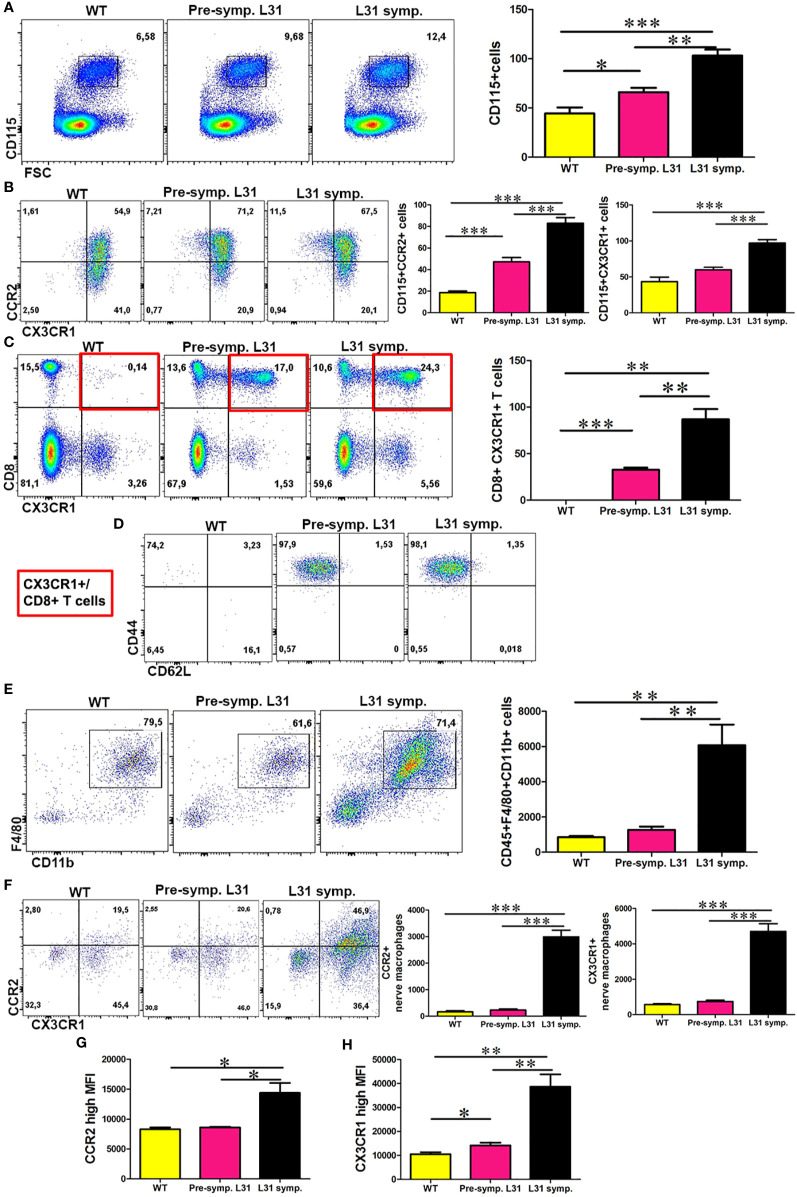
The expression of CCR2 and CX3CR1 in the blood and nerve of L31 mice. **(A)** Representative flow cytometry dot plot showed the number of monocytes in the blood of WT and L31 mice. Monocytes is significantly increased in L31 mice, with the highest increase found in L31 symptomatic mice. **(B)** A significant increase in CCR2 and CX3CR1 expressing monocytes mainly in the blood of L31-symptomartic mice. **(C)** Representative flow cytometry dot plot showed CX3CR1 is expressed only in the blood of L31 mice. **(D)** CX3CR1^+^ CD8^+^ T cells are of the effector memory phenotype (CD44^+^CD62L^-^). Red insert illustrates characterized cell population from C. **(E)** Macrophages are robustly increased in the nerve of L31 symptomatic mice. **(F)** A representative flow cytometry plot showed macrophages CCR2 and CX3CR1 expression in the nerve. A quantitative analysis showed that CCR2 and CX3CR1 are upregulated in L31 mice with the highest increase found in L31-symptomatic mice. **(G)** Quantitative analysis showed mean fluorescence intensity (MFI) of CCR2 expression in nerve macrophages of L31 mice. **(H)** Quantitative analysis showed MFI of CX3CR1 expression in nerve macrophages of L31 mice. Quantification in **(A–C)** depicted the number of cells per µl blood. Quantification in **(E, F)** depicted the number of cells per a segment of 2 cm long sciatic nerve. Pre-symptomatic refers to L31 mice without disease. L31-symptomatic refers to diseased L31 mice. Disease was induced by PSNL and experiments done 30 days post PSNL. n = 5-7/group; student’s *t* test; *p < 0.05; **p < 0.01; ***p < 0.001.

CCR2 has important roles in monocyte trafficking to PNS during inflammation (15), whereas CX3CR1 modulates macrophage activation and function (17). To address the potential involvement of CCR2 and CX3CR1 chemokine receptors in APN, we examined the expression of both receptors in blood monocytes and nerve macrophages in L31 mice. Flow cytometry analysis revealed a significant increase in the number of CD115^+^ blood monocytes in L31 mice as compared to WT controls (**Figure 1A**). We observed that the inflammatory subset (CCR2^+^) was significantly enhanced even before disease onset with the highest increase seen in diseased L31 mice (**Figure 1B**). Similarly, we observed an increased number of CX3CR1^+^ monocytes in L31 mice (**Figure 1B**). Flow cytometry analysis revealed that CX3CR1 was only expressed on CD8^+^ T cells in L31 mice which was further enhanced in diseased L31 mice (**Figure 1C**). All the CX3CR1 expressing CD8^+^ T cells in L31 mice were of the effector memory phenotype (CD44^+^CD62L^-^) (**Figure 1D**). In accordance with our previous reports (21), APN resulted in a massive increase in the number of CD45^+^F4/80^+^CD11b^+^ macrophages in sciatic nerves of diseased L31 mice (**Figure 1E**). A strong upregulation of both CCR2^+^ and CX3CR1^+^ subsets of nerve macrophages was observed in diseased L31 mice (**Figure 1F**). In addition to an increase in cell number, expression level within cells measured by mean fluorescence intensity (MFI) showed that both CCR2 and CX3CR1 expression on nerve macrophages was increased in diseased L31 mice. (**Figure 1G**). However, CX3CR1 MFI was even slightly higher in L31 mice before disease onset, and further upregulated in diseased nerves (**Figure 1H**). This data suggests a potential contribution of these two chemokine receptors in APN pathogenesis.

### CX3CR1 but Not CCR2 Deficiency Protects L31 Mice From Sensory and Motor Dysfunction

L31 mice spontaneously develop APN between 2-6 months. Mice exhibit limb weakness which rapidly progresses to paralysis and ultimately death (21). To understand the selective role of CCR2 and CX3CR1 chemokine receptors in APN, we backcrossed L31 mice with CCR2KO or CX3CR1KO mice and assessed the impact of CCR2 or CX3CR1 deficiency on mouse survival and neurological functions.

**Figure 2:**
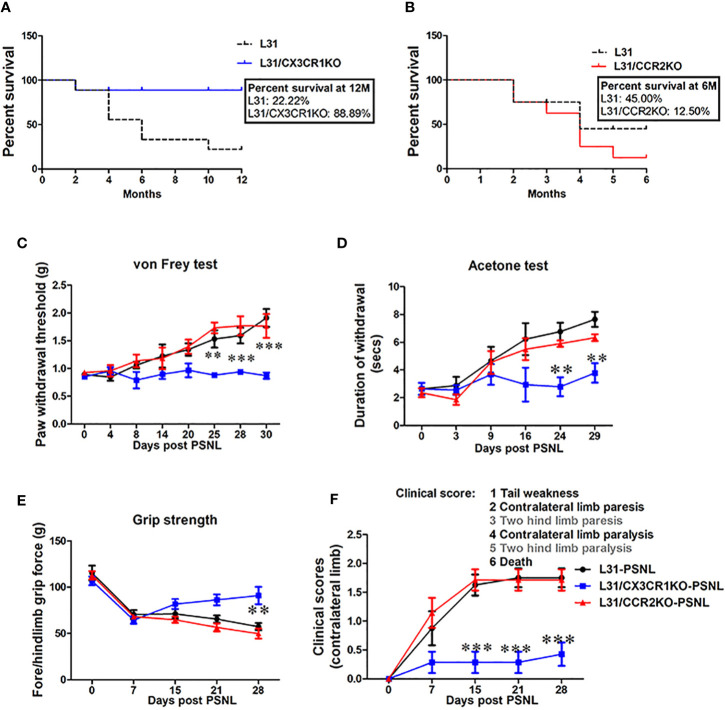
The effect of CCR2 or CX3CR1 deficiency on sensory and motor deficits in L31 mice. **(A)** Survival curve shows age of mice at death. Reduced survival was observed in L31 mice compared to L31/CX3CR1KO mice. **(B)** Survival curve shows slightly reduced survival in L31/CCR2KO mice compared to L31 mice. Survival curves were analyzed by Mantel-Cox test. **(C)** Paw withdrawal thresholds assessed using von Frey test showed mechanical hyposensitivity in L31 and L31/CCR2KO mice while in L31/CX3CR1KO mice, this was maintained to similar level of baseline, indicating that CX3CR1 deficiency prevented disease associated numbness/mechanical hyposensitivity. **(D)** Thermal sensitivity examined by acetone test was also normal only in L31/CX3CR1KO mice. **(E)** Quantitative data from grip strength showed that muscle strength was greater in L31/CX3CR1KO mice compared to L31 and L31/CCR2KO mice. **(F)** Clinical scores showed no appearance of neurological symptoms such as tail weakness and weakness of hind limb in L31/CX3CR1KO mice. n = 10-15/group; Two-way anova followed by Bonferroni’s test; **p < 0.01; ***p < 0.001.

As expected, L31 mice spontaneously developed disease and some of them died along the way, by the end of 12 months, only 2 over 10 mice survived (**Figures 2A, B**). However, none of L31/CX3CR1KO mice died of disease within 12 months (except one death for none-disease related reason) (**Figure 2A**). Surprisingly, when comparing survival rate of L31 mice to L31/CCR2KO mice, mice in both groups started to die at the age of 2 months, with more deaths observed in the L31/CCR2KO group (**Figure 2B**). By the age of 6 months, survival rate was 12.50% in L31/CCR2KO mice, and 45% in L31 mice (**Figure 2B**). CX3CR1KO and CCR2KO mice housed under the same conditions survive for at least one year without any disease phenotype, before they were used for experiments (data not shown).

While L31 mice spontaneously develop APN, an injury to a peripheral nerve in L31 mice before spontaneous onset accelerates the development of APN in other non-injured nerves (22). To further confirm disease incidence in CCR2 and CX3CR1 deficient L31 mice, we performed PSNL in L31, L31/CCR2KO and L31/CX3CR1KO mice at the age of 2–3 month, before their spontaneous onset, and evaluated sensory/motor nerve function of the contralateral limb. Overall, following PSNL surgery, all L31 and L31/CCR2KO, but none of L31/CX3CR1KO mice developed abnormal sensory and motor behavior (**Figures 2C–F**). L31 and L31/CCR2KO mice showed mechanical hyposensitivity in the contra limb from day 8 post-PSNL which gradually progressed until day 30 (**Figure 2C**). Paw withdrawal thresholds remained unchanged in L31/CX3CR1KO mice through the experimental period (**Figure 2C**). Similarly, acetone test revealed that cold allodynia was observed selectively in L31 and L31/CCR2KO mice (**Figure 2D**), but not in L31/CX3CR1KO mice, suggesting that CX3CR1 deficiency protects mice from sensory deficits. Furthermore, in the assessment of mouse motor functions, decreased grip strength was observed only in L31 and L31/CCR2KO mice (**Figure 2E**). This was further confirmed by hind limb weakness (increased clinical score) observed only in L31 and L31/CCR2KO mice, but not in L31/CX3CR1KO mice (**Figure 2F**). All these behavioral assessments further confirms that CX3CR1 deficient L31 mice are resistant to APN.

### CX3CR1 but Not CCR2 Deficiency Reduces the Expansion of Inflammatory Blood Monocytes and CD8^+^ T Cells in L31 Mice

**Figure 3:**
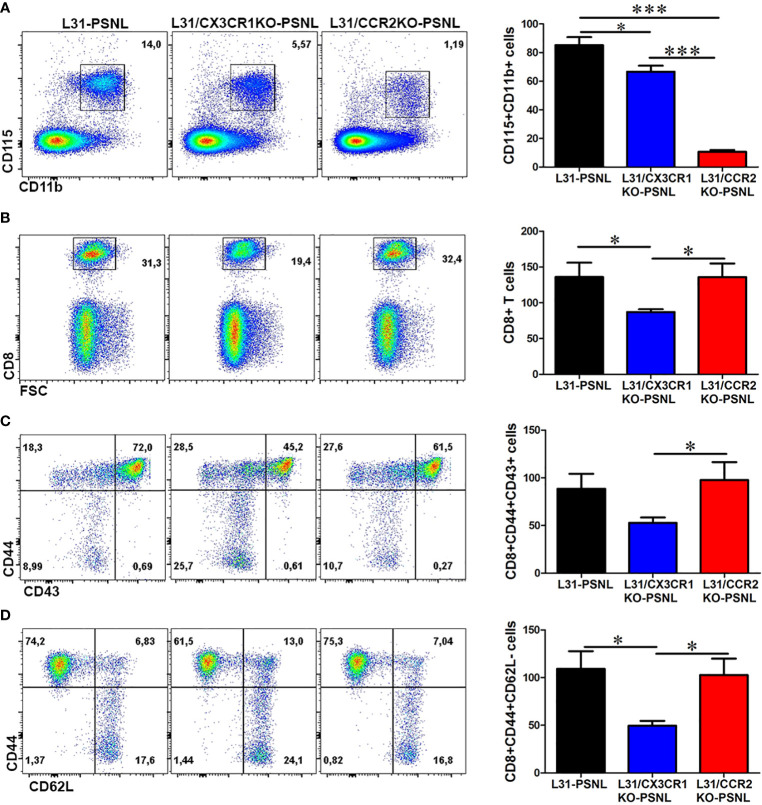
The effect of CCR2 and CX3CR1 deficiency on monocyte and CD8^+^ T cell activation status in the blood of L31 mice. **(A)** Representative flow cytometry dot plot and quantification analysis showed a decrease in the number of CD115^+^CD11b^+^ monocytes in the blood of L31/CX3CR1KO mice and an almost abolishment of CD115^+^CD11b^+^ monocytes in L31/CCR2KO mice. **(B)** Compared to L31 mice, the frequency and number of CD8^+^ T cells were reduced in the blood of L31/CX3CR1KO mice, but not affected in L31/CCR2KO mice **(C)** The majority of the reduced CD8^+^ T cells in L31/CX3CR1KO mice were of the activated subset (CD44^+^CD43^+^). **(D)** The frequency and number of CD8^+^ T cells with effector memory phenotype (CD44^+^CD62L^-^) were significantly reduced in the blood of L31/CX3CR1KO mice compared to L31 and L31/CCR2KO mice. Disease was induced by PSNL and experiments done 30 days post PSNL. All quantitative analyses are shown as number of cells per µl blood. n = 5-8/group; student’s *t* test; *p < 0.05; ***p < 0.001.

To better understand the contribution of CCR2 and CX3CR1 expression on disease development, we examined the number and phenotype of blood monocytes and CD8^+^ T cells in the blood of L31, L31/CX3CR1KO and L31/CCR2KO mice having PSNL. Comparing with L31 pre-symptomatic mice (**Figure 1A**), PSNL resulted in an increase of CD115^+^CD11b^+^ monocytes in L31 mice (**Figure 3A**), but not (or less) in L31/CX3CR1KO-PSNL mice. The number of monocytes in the blood of L31/CCR2KO-PSNL was dramatically decreased (**Figure 3A**), which might be attributed to the lack of CCR2, a crucial factor for monocyte egress from bone marrow. In addition, the number of CD8^+^ T cells in the blood of L31/CX3CR1KO-PSNL mice was significantly lower than those in L31-PSNL and L31/CCR2KO-PSNL mice (**Figure 3B**), as well as CD44^+^CD43^+^ and CD44^+^CD62L^-^ effector/memory CD8^+^ T cells (**Figures 3C, D**). These data suggest that loss of CX3CR1 attenuates systemic inflammation by reducing circulating effector cells.

### CX3CR1 but Not CCR2 Deficiency Significantly Suppresses Macrophages and CD8^+^ T Cells Mediated Inflammation in Sciatic Nerve of L31 Mice

**Figure 4:**
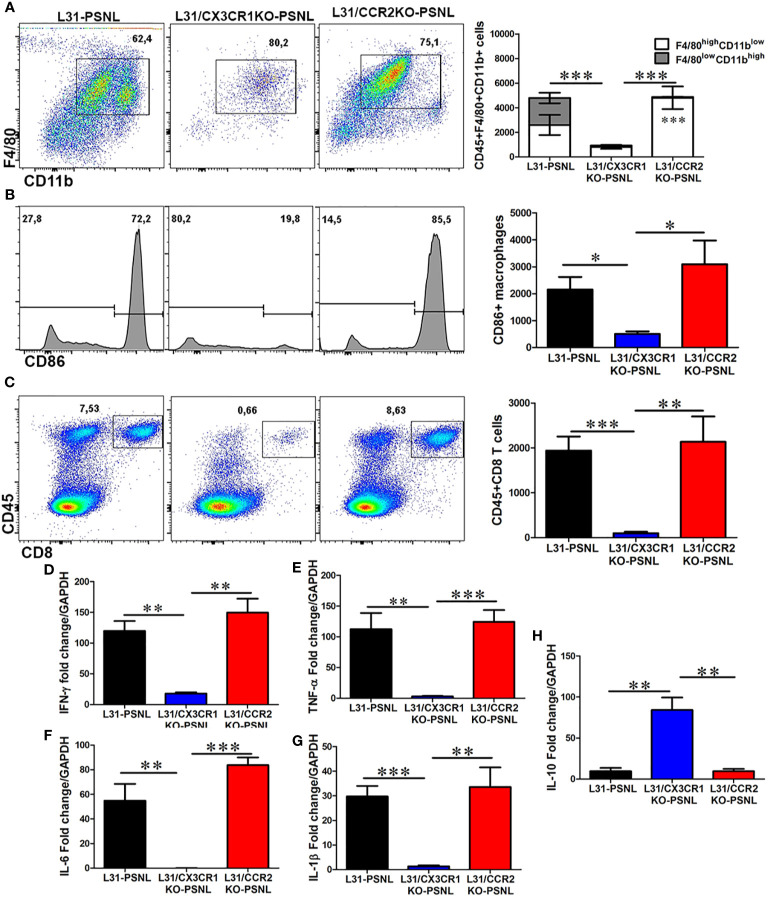
The effect of CCR2 and CX3CR1 deletion on macrophage and CD8^+^ T cell number, activation status and cytokine release in the nerve of L31 mice. **(A)** Quantitative analysis from flow cytometry showed a reduced number of macrophages (CD45^+^CD11b^+^F4/80^+^) in the nerve of L31/CX3CR1KO mice compared to L31 and L31/CCR2KO mice. Of note, two distinct subsets of macrophages were detected in L31 mice, which were distinguished by varying expression level of F4/80 and CD11b. Only one subset (F4/80^high^CD11b^low^) was seen in L31/CX3CR1KO and L31/CCR2KO mice, although the latter had significantly higher number. **(B)** The number of activated macrophages (CD86^+^) were significantly reduced in L31/CX3CR1KO mice. **(C)** Frequency and absolute number of infiltrating CD8^+^ T cells were significantly diminished in nerve of L31/CX3CR1KO mice. Real-time quantitative PCR showed a significantly reduced/undetectable expression of pro-inflammatory molecules **(D)** IFN-γ, **(E)** TNF-α, **(F)** IL-6, and **(G)** IL-1β, in sciatic nerve of L31/CX3CR1KO mice. **(H)** The mRNA level of the anti-inflammatory molecule, IL-10 was significantly enhanced in L31/CX3CR1KO mice. Disease was induced by PSNL and experiments done 30 days post PSNL. Quantification in a-c is shown as number of cells per a segment of 2 cm long sciatic nerve. n = 5-8/group; student’s *t* test; t*p < 0.05; **p < 0.01; ***p < 0.001.

Macrophage and CD8^+^ T cell mediated local inflammation is essential for demyelination and axonal damage in the nerve. The number and phenotype of these effector cells were assessed in sciatic nerves, contralateral to the PSNL, of all mice with three different genetic background. As previously reported (21), CD45^+^F4/80^+^CD11b^+^ macrophages was found to abundantly accumulate in sciatic nerve of L31-PSNL mice (**Figure 4A**), which was not the case in L31/CX3CR1KO-PSNL mice (**Figure 4A**). However, despite a dramatic decrease of circulating monocytes in L31/CCR2KO-PSNL mice, nerve macrophages in L31/CCR2KO-PSNL mice were expanded to the similar level of that in L31-PSNL mice (**Figure 4A**). It is interesting to note that nerve macrophages in L31-PSNL mice were composed of two subsets, F4/80^high^CD11b^low^ and F4/80^low^CD11b^high^ cells, reflecting respectively resident and newly recruited blood derived macrophages; while only one subset, F4/80^high^CD11b^low^ resident macrophages observed in L31/CCR2KO-PSNL mice (**Figure 4A**), suggesting that CCR2 deficiency prevented monocyte trafficking into the nerve. As a sign of macrophage activation, B7.2 (CD86) expression was significantly higher in L31-PSNL and L31/CCR2KO-PSNL mice than that in L31/CX3CR1-PSNL mice (**Figure 4B**). In parallel to an increase of activated macrophages in L31-PSNL and L31/CCR2KO-PSNL mice, we observed a massive number of infiltrating CD8^+^ T cells in the sciatic nerve of both groups of mice, with a few CD8^+^ T cells seen in the nerve of L31/CX3CR1KO-PSNL mice (**Figure 4C**).

Furthermore, consistent with our previous studies (19, 22), mRNA levels of IFN-γ (**Figure 4D**), TNF-α (**Figure 4E**), IL-6 (**Figure 4F**), and IL-1β (**Figure 4G**), were strikingly high in sciatic nerves of L31-PSNL and L31/CCR2KO-PSNL mice, suggesting that changes in the number of effector cells described above (**Figures 4A–C**) were associated with the release of functional mediators. Similar increase was not observed in the nerve of L31/CX3CR1KO-PSNL mice. In addition, a significantly increased expression of anti-inflammatory cytokine, IL-10 was observed in L31/CX3CR1KO-PSNL mice but not in L31-PSNL and L31/CCR2KO-PSNL mice (**Figure 4H**). Collectively, the above data indicates that CX3CR1, but not CCR2 signaling has an important role in regulating macrophage and CD8^+^ T cell mediated inflammatory response.

### Myelin and Axon Integrity Are Preserved in L31/CX3CR1KO Mice but Not in L31/CCR2 KO Mice

**Figure 5:**
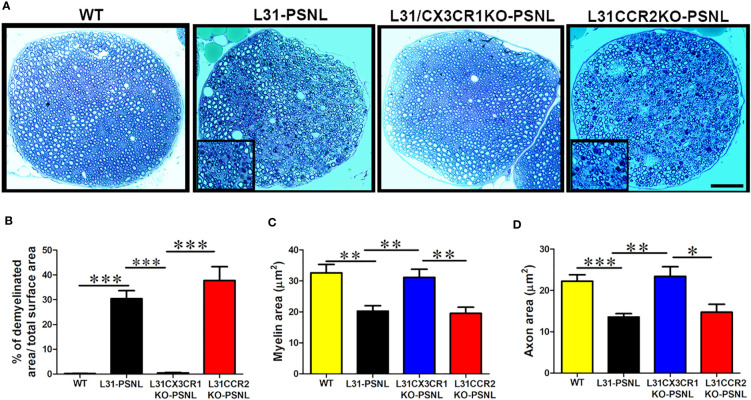
The effect of CCR2 and CX3CR1 deficiency on nerve structure in L31 mice. **(A)** Representative micrographs of sciatic nerve cross-sections showed partial axonal damage and myelin loss in L31 and L31/CCR2KO mice, which was absent in L31/CX3CR1KO mice. Nerve structure in L31/CX3CR1KO remained comparable to WT mice. Inserts illustrated immune cell infiltration with roundish dark blue staining. **(B)** Quantitative analysis revealed a partial axonal and myelin loss (30%) in L31 mice and (40%) in L31/CCR2KO mice, which was prevented in L31/CX3CR1KO mice; **(C, D)** Assessment of myelin and axon area on randomly selected 100 myelinated axons revealed that in the area where nerve structure seemed preserved in L31 and L31/CCR2KO mice, myelin and axon area were reduced indicating that myelin could become thinner and axons could be atrophic, which was prevented in L31/CX3CR1KO mice. Disease was induced by PSNL and experiments done 30 days post PSNL. n = 5-7/group; student’s *t* test; *p < 0.05; **p < 0.01; ***p < 0.001.

Demyelination and/or axonal loss are the pathological hallmark in GBS patients and also seen in diseased L31 and L31/CD4KO mice (21, 25). In addition to functional outcomes and inflammatory reaction, we also assessed nerve tissue integrity with histological analysis of myelin and axons in sciatic nerves. Nerves in toluidine blue stained, semi-thin plastic embedded sections displayed a well-organized nerve structure with pale round axons of different sizes in WT mice (**Figure 5A**). However, in L31 and L31/CCR2KO mice, there were severe axonal loss and demyelination with cell infiltration in the nerve contralateral to the PSNL (**Figure 5A**). Inserts in **Figure 5A** displayed infiltrated immune cells with dark blue staining. Demyelinated area relative to total endoneurial surface was about 30% and 40%, respectively, in L31 mice and L31/CCR2KO mice (**Figure 5B**). Furthermore, in the area where the nerve structure looked unscathed and relatively healthy, quantitative analysis of randomly selected 100 myelinated axons revealed a reduction in myelin and axon area (**Figures 5C, D**), indicating a potential demyelination and axonal atrophy in these areas as well. Both representative histology images (**Figure 5A**) and quantitative assessments (**Figures 5B–D**) clearly demonstrated a fairly well-preserved nerve structure in the contralateral nerve of L31/CX3CR1KO-PSNL mice (**Figure 5**), that was accompanied by an absence of systemic (**Figure 3**) and local inflammation (**Figure 4**), further confirming the protective effect of CX3CR1 deficiency.

### Distinct Roles of CX3CR1 and CCR2 in Monocyte/Macrophage and CD8^+^ T Cell Activation Leads to Different Functional Outcomes in APN

**Figure 6:**
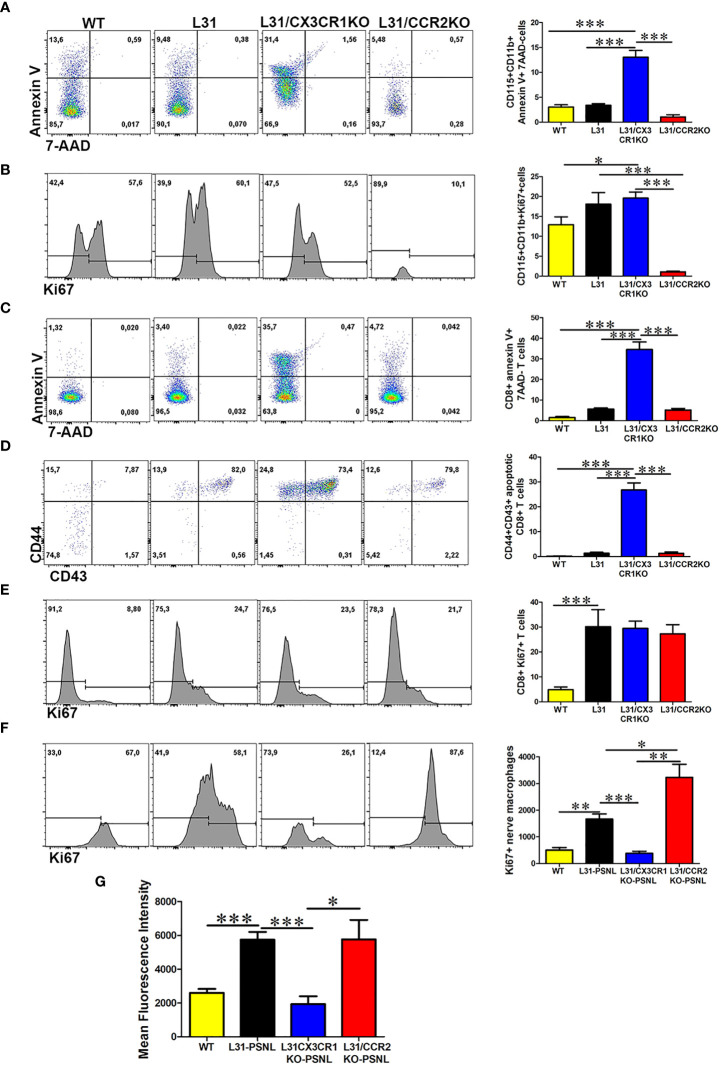
The impact of CX3CR1 and CCR2 deficiency on monocyte/macrophage and CD8^+^ T cells survival and function in L31 mice. **(A)** Representative flow cytometry plot and quantitative analysis of monocytes apoptosis. Compared to L31 and L31/CCR2KO mice, increased monocyte death was observed in the blood of L31/CX3CR1KO mice. **(B)** Similar level of cell proliferation was seen in L31 and L31/CX3CR1KO mice, indicating that no enhanced proliferation occurred to compensate the increased cell death. Monocyte cell proliferation was barely detectable in L31/CCR2KO mice. **(C)** CD8^+^ T cells apoptosis (Annexin V^+^/7-AAD^-^) was observed essentially in L31/CX3CR1KO mice. **(D)** The majority of apoptotic CD8^+^ T cells were of the activated phenotype (CD44^+^CD43^+^). **(E)** Proliferation of CD8^+^ T cells was similar in all three groups, L31, L31/CX3CR1KO and L31/CCR2KO mice, showing no enhanced proliferation to compensate for CD8^+^ T cell death in L31/CX3CR1KO mice. **(F)** Macrophage proliferation was strongly enhanced in L31/CCR2KO mice, 87% (Ki67^+^ cells over total nerve macrophages (F4/80CD11b) in L31/CCR2KO mice and 58% in L31 mice. **(G)** mean fluorescent intensity (MFI) depicts the amount of phagocytosed beads by nerve macrophages. The highest MFI was observed in L31and L31/CCR2KO mice, which was significantly reduced in L31/CX3CR1KO mice. Quantification in A-E depicted the number of cells per µl blood. Quantification in F depicted the number of cells per a segment of 2 cm long sciatic nerve. Disease was induced by PSNL and experiments done 30 days post PSNL. n = 5-6/group; student’s *t* test; *p < 0.05; **p < 0.01; ***p < 0.001.

The fact that CX3CR1 but not CCR2 deficiency protected L31 mice from APN intrigued us to further investigate the underlying mechanisms. In the blood, monocyte cell death (CD115^+^CD11b^+^Annexin V^+^7ADD^-^ cells) was significantly higher in CX3CR1 deficient mice, (**Figure 6A**). At the same time, comparing with WT mice, monocyte cell proliferation (CD115^+^CD11b^+^Ki67^+^ cells) increased almost equally in L31 and L31/CX3CR1KO mice, although in L31/CCR2KO mice, monocyte proliferation was absent, as lack of CCR2 expression resulted in an almost complete abolishment of circulating monocytes (**Figure 6B**). Interestingly, we also observed a significant number of CD8^+^ T cells undergoing cell death in L31/CX3CR1KO mice (**Figure 6C**), and the majority of dying CD8^+^ T cells are CD44^+^/CD43^+^ activated CD8^+^ T cells (**Figure 6D**). CD8^+^ T cell proliferation (CD8^+^Ki67^+^ cells) remained comparable between L31, L31/CX3CR1KO and L31/CCR2KO mice (**Figure 6E**). This result suggests that CX3CR1 is crucial for the survival of monocytes and CD8^+^ T cells. There was no evidence of increased cell proliferation, neither monocyte, nor CD8^+^ T cell in the circulation, to compensate for CX3CR1 deficiency-associated cell death. Selective expression of CX3CR1 on activated CD8^+^ T cells (**Figures 1C, D**) led us to assume that CX3CR1 deficiency contributes essentially to the reducing activated CD8^+^ T cells, a required player in the genesis of APN in L31 mice (**Figure 3C**).

Additionally, our *ex vivo* phagocytosis assay revealed an impact of CX3CR1 deficiency on macrophage phagocytic ability. Fluorescent beads engulfed by nerve macrophages in L31/CX3CR1KO mice was at the level of WT mice, and which was significantly reduced compared to L31 mice (**Figure 6G**), while enhanced macrophage phagocytic activity has been considered crucial in APN pathogenesis.

Although CCR2 deficiency almost abolished circulating monocytes, it failed in preventing APN in L31 mice. To compare with L31 and L31/CX3CR1KO mice, we noticed that CCR2 is required to maintain blood monocyte population, but not CD8^+^ T cell population. The number of annexin V^+^/7AAD^-^ apoptotic monocytes and CD8^+^ T cells in L31/CCR2 KO mice were comparable to L31 mice in their respective compartments (**Figures 6A, C**). In the nerves, while blood derived F4/80^low^CD11b^high^ macrophage subset was absent in L31/CCR2KO mice (**Figure 4A**), the number of Ki67^+^ macrophages were much higher in sciatic nerves of L31/CCR2KO mice than that in L31/CX3CR1KO mice, even significantly higher than that in L31 mice (**Figure 6F**). The increase of macrophage cell proliferation in L31/CCR2KO mice compensated the lack of blood derived subset, leading to an accumulation of macrophages, which is sufficient, in the presence of activated CD8^+^ T cells (**Figure 4C**), to generate inflammatory reaction to damage the nerve. The lack of CCR2 expression did not affect macrophage phagocytic ability (**Figure 6G**).

## Discussion

Autoimmune peripheral neuropathy is a group of immune-mediated disorders affecting the peripheral nervous system. Disease progression relies on an inflammatory response, predominated by macrophage and T cell infiltration and activation. Understanding essential functions of these immune cells and their regulatory mechanisms are important for developing effective therapeutic approaches to promote complete recovery. We report here that chemokine receptors CCR2 and CX3CR1 contribute differently to disease in L31 mice. CX3CR1 is required in initiating macrophage and CD8^+^ T cell mediated-autoimmune inflammatory response in peripheral nerves. CX3CR1 deficiency confers neuroprotection and prevents loss of motor/sensory function. Our results indicate that CX3CR1 is needed for maintaining circulating monocyte and CD8^+^ T cell survival. While migration of a significant number of activated CD8^+^ T cells to peripheral nerves is essential in autoimmune response in nerve, recruitment of monocytes into PNS seems optional. CCR2 mediated monocyte trafficking can be replaced by local expansion of nerve macrophages, where enhanced macrophage phagocytosis is sustained by the presence of CX3CR1. CCR2 deficiency failed in preventing APN in L31 mice.

Two subsets of blood monocytes with varying levels of CCR2 and CX3CR1 have been described (13). CCR2^-^CX3CR1^+^ monocytes have a longer half-life and mediate patrolling of monocytes in the vascular space under steady state. They may contribute to certain tissue macrophages, when needed. Conversely, the CCR2^+^CX3CR1^-^ subset, with a short half-life, mediates rapid recruitment of monocytes into inflamed tissue (26). Although GBS is an organ/tissue specific autoimmune disorder, systemic inflammation has been seen in patients (27) and in experimental animals, e.g., EAN (28). Compared with healthy control, the number of monocytes is higher in GBS patients (29). An enhanced CCR2 expression has been observed in the blood of both GBS patients and EAN rats (8, 9, 12). Our investigation with L31 mice also depicted an increase of monocytes in blood, even before disease onset, this was further enhanced when mice became symptomatic. We observed that both CCR2^+^ and CX3CR1^+^ subsets were expanding in the blood of diseased L31 mice. Both CCR2 and CX3CR1 contributed to maintain this increased monocyte pool in L31 mice. By preventing monocyte egress from the bone marrow (30, 31), CCR2 deficiency led to an almost complete depletion of circulating monocytes in L31 mice. Confirming previous observation that CX3CR1 is crucial for the survival of blood monocytes (16), we found that although much less important, the number of monocytes in L31/CX3CR1KO mice was also significantly reduced, which could be attributed to an increase in cell death.

While CX3CR1 is widely distributed in myeloid cells, recent studies have implicated CX3CR1 expression on CD8+ T cell function. CX3CR1 defines three antigen-experienced CD8+ T cell subsets with distinct roles in immune surveillance and homeostasis. CX3CR1hi cells have been found overlapped with classically defined effector/memory CD8+ T (CD8+ TEM) cells (32), which are indeed, the majority of CD8+ T cells found in L31 mice, and they are critical effectors in disease. CX3CR1 expression also discriminates terminally differentiated cytotoxic effector cells, which are ready to infiltrate into inflamed tissues (33, 34). CX3CR1 signaling may aid CD8+ T cell survival by suppressing the transcription of the apoptotic molecule bmf (34). In L31 mice, CX3CR1 deficiency resulted in a decrease of CD8+ T cells, more specially CD44+CD43+ and CD44+CD62L- CD8+ T cells, in the blood. This could be attributed to the selective CX3CR1 expression on CD8+ TEM cells and the requirement of CX3CR1 on CD8+ T cell survival. Although CD8+ T cells can also express CCR2 (35), it appears that lacking CCR2 expression did not affect CD8+ T cell survival, the number of total CD8+ T cells, as well as CD8+ TEM in L31/CCR2KO mice were similar to that of L31 mice. We have previously reported that in L31 mice, CD8+ T cells are pathogenic, activated CD8+ T cells, more specifically effector/memory phenotype, are mandatory in developing APN (22, 36). We also reported that in L31 mice lacking CD4+ T cells, disease process is accelerated, mice experience demyelination and axonal loss, indicating an immunoregulatory role for CD4+ T cells in disease pathogenesis in L31 mice (21, 37). Decreasing circulating CD8+ TEM cells could be one of the major underlying mechanisms that CX3CR1 deficiency prevents APN in L31 mice.

Macrophages are the main effector cells in peripheral neuropathies, no matter if it is autoimmune disorder by nature, or triggered by trauma, metabolic dysfunction and/or genetic mutation involving secondary inflammation. A recent transcriptomic characterization of peripheral nerve macrophages demonstrated that following nerve injury, blood derived macrophages exhibit anti-inflammatory properties immediately after entering the nerve (38). Few days after, these recruited macrophages fully adapt to the nerve environment and upregulate M1-like markers (38). This study further shows that in the steady state, endoneurial resident macrophages express activated genes which are crucial in sensing their environment (38, 39). In L31 mice, abundant macrophages accumulated in diseased nerves, which was composed of F4/80^high^CD11b^low^ resident macrophages and F4/80^low^CD11b^high^ blood-derived macrophages. It is plausible that upon entry of blood-derived macrophages into the nerve, there exists a crosstalk between resident and newly recruited macrophages, which orchestrates the composition and function of entire pool of nerve macrophages. When circulating monocytes were abolished in L31 mice lacking CCR2 expression, F4/80^low^CD11b^high^ blood-derived macrophages were deprived, resident macrophages were capable of sensing the absence of their blood derived partners. A significant increase of resident macrophage proliferation was detected which made up for the loss of their blood-derived counterpart. These newly generated resident macrophages were totally functional. Same as those in L31 mice, they expressed high level of CD86, released essential inflammatory mediators, such as TNF-α, IL-1β and IL-6, and they maintained phagocytic function. However, nerve macrophage phagocytosis was severely impaired in L31/CX3CR1KO mice, indicating that the contribution of CX3CR1^+^ macrophages to APN in L31 mice could also be endorsed by its role in phagocytosis. Deletion of CX3CR1 could impede phagocytosis which has been reported for microglia in cuprizone-fed mice (40), cultured bone marrow-derived macrophages (41) and intramuscular macrophages (42). Overall, this compensatory mechanism argues that intrinsic resident macrophages could be sufficient to interact with infiltrated CD8^+^ T cells to promote APN in L31 mice. Critical contribution of resident macrophages to peripheral neuropathy are not limited to autoimmune origin, such as in L31 mice and EAN rats (43), but also observed in models of trauma (44, 45) and hereditary neuropathy where macrophage response is mainly of intrinsic origin (46). Usually, resident macrophages exhibit rapid activation, enhanced phagocytosis, and proliferation before the entry of their blood derived counterparts (43).

## Conclusion

**Figure 7:**
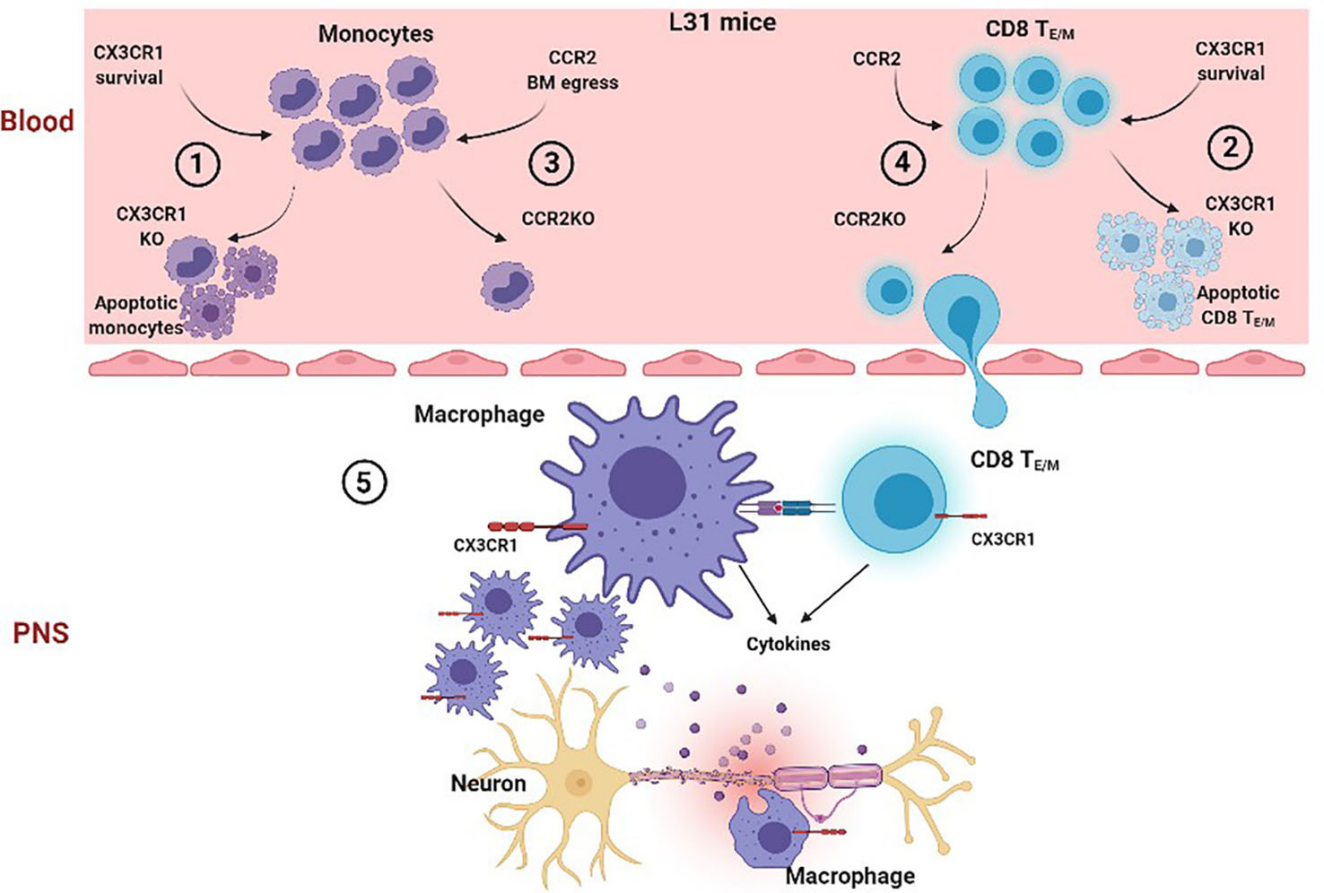
Proposed cascade of CCR2 and CX3CR1 mediated mechanisms in the generation of APN in L31 mice. In the blood, when CX3CR1 is absent, a significant number of circulating monocyte (1) and effector/memory CD8^+^ T cells (2) undergo apoptosis, suggesting that CX3CR1 is required for monocyte and activated CD8^+^ T cell survival. However, when CCR2 is absent, although the number of circulating monocytes drops dramatically (3) due to reduced bone marrow egress, CD8^+^ T_E/M_ survival/function is not affected. They are able to migrate into peripheral nerves in L31 mice (4). In peripheral nerves, although lack of CCR2 prevents blood derived macrophage recruitment, resident CX3CR1^+^ macrophages proliferate abundantly to make up an activated macrophage population, which has enhanced phagocytic ability, They communicate with infiltrated CD8^+^ T_E/M_, resulting in inflammatory demyelinating peripheral neuropathy (5). However, CX3CR1 deficiency associated CD8^+^ T_E/M_ cell death dramatically reduces the number of activated CD8^+^ T cells, preventing a successful autoimmune response and preserving nerve integrity. Thus, CX3CR1 expression on CD8^+^ T cells is crucial in pathogenesis in L31 mice.

Distinctive contribution of CX3CR1 and CCR2 in APN is summarized in **Figure 7**. The study provided clear evidence that in L31 mice 1) Chemokine receptors CX3CR1 and CCR2 contribute differently to the development of APN; loss of CX3CR1 but not CCR2 expression protects L31 mice from inflammatory peripheral neuropathy and neurological dysfunction; 2) Disease genesis is independent of CCR2 mediated blood-derived macrophage recruitment, which can be replaced by compensatory proliferation of resident macrophages in peripheral nerve; 3) CX3CR1 could contribute to APN *via* its critical involvement in maintaining the survival of circulating effector/memory CD8^+^ T cells, as well as phagocytic ability of nerve macrophages, two mandatory components in generating APN. Our findings highlighted the requirement of CX3CR1 in the pathogenesis of APN in L31 mice and potential underlying mechanisms. We propose that blocking CX3CR1 signaling could be an interesting therapeutic strategy to consider for GBS patients. It has potential to limit inflammatory response and protect nervous tissue.

## Data Availability Statement

The raw data supporting the conclusions of this article will be made available by the authors, without undue reservation.

## Ethics Statement

All procedures were in accordance with the guidelines of the Canadian Council on Animal Care and approved by the animal care committee of McGill University.

## Author Contributions

OO and XS performed experiments. OO, SF, and JZ designed the study, wrote the manuscript. All authors contributed to the article and approved the submitted version.

## Funding

This work was supported by funding from the Canadian Institutes for Health Research (CIHR) PJT-155929, the Louise and Alan Edwards Foundation to JZ.

## Conflict of Interest

The authors declare that the research was conducted in the absence of any commercial or financial relationships that could be construed as a potential conflict of interest.

## Publisher’s Note

All claims expressed in this article are solely those of the authors and do not necessarily represent those of their affiliated organizations, or those of the publisher, the editors and the reviewers. Any product that may be evaluated in this article, or claim that may be made by its manufacturer, is not guaranteed or endorsed by the publisher.

## Abbreviations

APN, autoimmune peripheral neuropathy, GBS, Guillain Barre Syndrome, PNS, peripheral nervous system, EAN, experimental allergic neuritis, PSNL, partial sciatic nerve ligation, CSF, cerebrospinal fluid, PFA, paraformaldehyde, ACK lysing buffer, Ammonium-Chloride-Potassium lysing buffer, qPCR, quantitative PCR, GAPDH, Glyceraldehyde 3-phosphate dehydrogenase, WT, wild type, MFI, mean fluorescence intensity.
