# Corrigendum: CX3CR1 But Not CCR2 Expression Is Required for the Development of Autoimmune Peripheral Neuropathy in Mice

**DOI:** 10.3389/fimmu.2021.765892

**Published:** 2021-09-24

**Authors:** Oladayo Oladiran, Xiang Qun Shi, Sylvie Fournier, Ji Zhang

**Affiliations:** ^1^ The Alan Edwards Centre for Research on Pain, McGill University, Montreal, QC, Canada; ^2^ Department of Microbiology & Immunology, McGill University, Montreal, QC, Canada; ^3^ Department of Neurology & Neurosurgery, McGill University, Montreal, QC, Canada; ^4^ Faculty of Dentistry, McGill University, Montreal, QC, Canada

**Keywords:** macrophages, CD8^+^ T cells, autoimmune peripheral neuropathy, CX3CR1, CCR2, apoptosis, phagocytosis

In the original article, there was a mistake in **Figure 6** as published. The wrong representative FACS plot was inserted for L31/CCR2KO in **Figure 6A**. The corrected **Figure 6** appears below.

**Figure 6:**
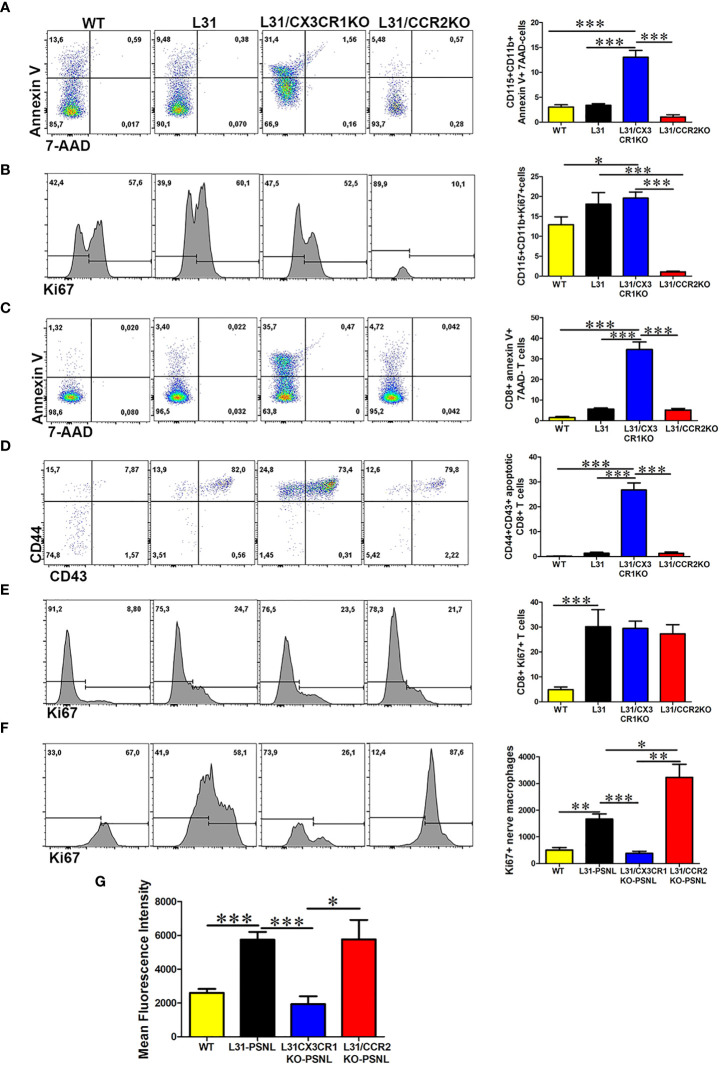
The impact of CX3CR1 and CCR2 deficiency on monocyte/macrophage and CD8+ T cells survival and function in L31 mice. **(A)** Representative flow cytometry plot and quantitative analysis of monocytes apoptosis. Compared to L31 and L31/CCR2KO mice, increased monocyte death was observed in the blood of L31/CX3CR1KO mice. **(B)** Similar level of cell proliferation was seen in L31 and L31/CX3CR1KO mice, indicating that no enhanced proliferation occurred to compensate the increased cell death. Monocyte cell proliferation was barely detectable in L31/CCR2KO mice. **(C)** CD8+ T cells apoptosis (Annexin V+/7-AAD-) was observed essentially in L31/CX3CR1KO mice. **(D)** The majority of apoptotic CD8+ T cells were of the activated phenotype (CD44+CD43+). **(E)** Proliferation of CD8+ T cells was similar in all three groups, L31, L31/CX3CR1KO and L31/CCR2KO mice, showing no enhanced proliferation to compensate for CD8+ T cell death in L31/CX3CR1KO mice. **(F)** Macrophage proliferation was strongly enhanced in L31/CCR2KO mice, 87% (Ki67+ cells over total nerve macrophages (F4/80CD11b) in L31/CCR2KO mice and 58% in L31 mice. **(G)** mean fluorescent intensity (MFI) depicts the amount of phagocytosed beads by nerve macrophages. The highest MFI was observed in L31and L31/CCR2KO mice, which was significantly reduced in L31/CX3CR1KO mice. Quantification in A-E depicted the number of cells per µl blood. Quantification in F depicted the number of cells per a segment of 2 cm long sciatic nerve. Disease was induced by PSNL, and experiments done 30 days post PSNL. n=5-6/group; student’s t test; *p < 0.05; **p < 0.01; ***p < 0.001.

The authors apologize for this error and state that this does not change the scientific conclusions of the article in any way. The original article has been updated.

## Publisher’s Note

All claims expressed in this article are solely those of the authors and do not necessarily represent those of their affiliated organizations, or those of the publisher, the editors and the reviewers. Any product that may be evaluated in this article, or claim that may be made by its manufacturer, is not guaranteed or endorsed by the publisher.

